# TrainSel: An R Package for Selection of Training Populations

**DOI:** 10.3389/fgene.2021.655287

**Published:** 2021-05-07

**Authors:** Deniz Akdemir, Simon Rio, Julio Isidro y Sánchez

**Affiliations:** ^1^Agriculture & Food Science Centre, Animal and Crop Science Division, University College Dublin, Dublin, Ireland; ^2^Centro de Biotecnologia y Genómica de Plantas (CBGP, UPM-INIA), Instituto Nacional de Investigación y Tecnologia Agraria y Alimentaria (INIA), Universidad Politécnica de Madrid (UPM), Madrid, Spain

**Keywords:** training optimization, machine learning, genomic selection, genomic prediction, image classification, multi-objective optimization, mixed models

## Abstract

A major barrier to the wider use of supervised learning in emerging applications, such as genomic selection, is the lack of sufficient and representative labeled data to train prediction models. The amount and quality of labeled training data in many applications is usually limited and therefore careful selection of the training examples to be labeled can be useful for improving the accuracies in predictive learning tasks. In this paper, we present an R package, TrainSel, which provides flexible, efficient, and easy-to-use tools that can be used for the selection of training populations (STP). We illustrate its use, performance, and potentials in four different supervised learning applications within and outside of the plant breeding area.

## 1. Introduction

Genomic selection (GS) uses supervised learning for predicting genetic values of phenotyped and un-phenotyped individuals by using genomewide molecular markers (Meuwissen et al., 2001). Genomic prediction (GP) models are built using a training data, i.e., genomic and phenotypic data for a set of individuals. Unfortunately, phenotyping of plants is an expensive and time-consuming process due to factors such as reliance on human input and budget time and resource constraints. Therefore, the most important current bottleneck in application of GS in plant breeding programs is phenotyping. Selection of training populations (STP) in this context refers to identification of a set of training individuals to be phenotyped.

While the usefulness of optimal training set (TRS) in GS is clearly supported by the literature (Rincent et al., 2012; Akdemir et al., 2015; Isidro et al., 2015; Lorenz and Smith, 2015; He et al., 2016; Cericola et al., 2017; Neyhart et al., 2017; Norman et al., 2018; Akdemir and Isidro-Sánchez, 2019; Guo et al., 2019; Mangin et al., 2019; de Bem Oliveira et al., 2020; Olatoye et al., 2020; Yu et al., 2020; Kadam et al., 2021), the flexible and efficient software tools for implementing them have been limited. Indeed, only a few software tools such as STPGA (Akdemir, 2017) and TSDFGS (Ou and Liao, 2019) are available for public use. The TSDFGS is an R package that focuses on optimization of the TRS by a genetic algorithm (GA) and can be used for STP based on three built-in design criteria. Similarly, STPGA is an R package that uses a modified GA for solving subset selection problems but also allows users to chose from many predefined or user-defined criteria. Here, we designed a TrainSel package that provides many more options, for example, the ability to select multiple sets from multiple candidate sets, specification of whether or not the resulting set needs to be ordered, or the power to perform multi-objective optimization. In addition, TrainSel can be used for searching for solutions to variety of TRS and experimental design problems, such as randomized complete block design, lattice design, etc. TrainSel uses GA in conjunction with simulated annealing (SA) steps, and functions are written in C++ using Rcpp (Eddelbuettel et al., 2011), and therefore, improves performance and is more efficient compared to both of the above alternatives.

In addition, the TrainSel package was designed to be applied not just for genomic assisted breeding situations, it can also be utilized for STP in general supervised learning problems. Supervised learning refers to the exercise of building predictive models that allow us to predict the states of certain output variables (referred as labels) based on certain input variables. To build supervised learning models we make use of a training dataset that includes observations of both the input variables and the labels, and generally, the larger and more representative the training dataset, the greater is the statistical power for supervised learning. We use the term label throughout this article to refer to the output variables that we are trying to predict. In genomic selection, labeling a genotype refers to measurement of phenotypic values for that genotype in one or more environments.

In this paper, we demonstrated the usage of the TrainSel R package for STP on genomic assisted breeding applications, but also included other applications to illustrate that STP may also be worthwhile for other supervised learning tasks, such as image classification.

## 2. Materials and Methods

### 2.1. Populations for Selection of Training Population (STP)

**Figure 1:**
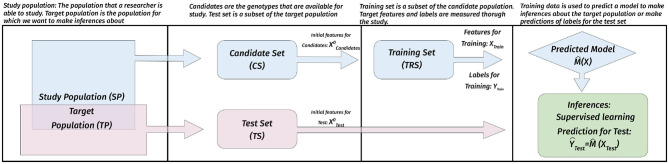
Populations in STP and their use. The study population is the population that is accessible to the researcher. The candidate set (CS) is a countably finite representative subset of the SP, similarly, the test set (TS) is a countably finite representative subset of the target population. The initial information about the topology of the CS and TS ($X_{Candidate}^{0}$ and $X_{Candidate}^{0}$) is used to identify a subset of the CS as the training set (TRS) for measuring the labels (phenotypic values in GS) (*Y*_*Train*_) and other related features (*X*_*Train*_) (for instance, environmental covariates). The training data for TRS is used to build supervised learning models which are then used to make inferences and predictions.

During STP, we will encounter different types of populations. The target population (Akdemir and Isidro-Sánchez, 2019) is the population that the researcher is interested in, i.e., the population we want to make inferences about. The study population is the population that is accessible to the researcher. The candidate set (CS) is a countably finite representative subset of the study population, similarly, the test set (TS) is a countably finite representative subset of the target population. We assume that we either have an idea about the topology (referring to the initial data available on CS and TS before doing the experiment) of the union of the CS and TS, or that it is relatively easy to obtain this information. Finally, the initial information about the topology of the CS and TS is used to identify a subset of the CS as the training set (TRS) for measuring the labels and additional features. These populations and the default supervised learning paradigm is illustrated in Figure 1.

### 2.2. Optimization Algorithm in TrainSel

Selection of training population involves the selection of a subset from a set of candidates and therefore is a combinatorial problem. These problems are typically exponential in terms of computational complexity and may require exploring all possible solutions. Nevertheless, many modern publications point to the effectiveness of applying metaheuristics in obtaining “good” answers to combinatorial optimization problems.

TrainSel uses a combination of GA (Holland, 1992) and simulated annealing (SA) algorithm (Haines, 1987) for solving combinatorial optimization problems. Genetic algorithm uses techniques inspired by natural evolution such as inheritance, mutation, selection, and crossover to generate better solutions through iterations (Holland, 1992). Simulated annealing moves between solutions using a perturbation and acceptance scheme. At each iteration, a new solution is generated by perturbing the current solution, and this new solution is accepted if it improves the optimization criterion. If the perturbed solution is inferior to the current solution the new solution is accepted based on an acceptance probability that is inversely proportional to the distance of the new solution to the current solution and the current temperature of the system (Haines, 1987). Temperature parameter varies during the iterations of the SA algorithm and usually is a decreasing function of the iteration number. Acceptance of inferior solutions during the SA iterations allows the algorithm to explore more of the possible space of solutions.

Algorithms such as GA and SA outperform other traditional methods in many applications, as they are flexible and easy to implement (no mathematical analysis is needed when considering a large, complex, non-smooth, poorly-understood optimization problem). There is no proof of convergence for either GA or SA, however, they are effective on a large range of classic optimization problems, and more specifically, have proved to be effective for approximating globally optimal solutions to many combinatorial optimization problems (Glover and Kochenberger, 2006; Fischetti and Lodi, 2010).

Algorithm 1 describes the main steps of the sample selection algorithm for the single optimization criteria problems. A similar algorithm is used when optimizing more than one criteria. The main difference is that the elite solutions of a population are defined as the non-dominated solutions of the current population.

**Algorithm 1:** Combinatorial optimization algorithm in TrainSel

1: *t* = 0.
2: Initialization—Create an initial population of solutions of desired size, *S*_*t*_. Parameters: npop
3: **repeat**
4: *t* = *t* + 1.
5: *S*_*t*_ = ∅.
6: Selection—Identify the best solutions in *S*_*t*−1_ by the ordering of criterion values. Let the best solutions be *s*_*t*_. Parameters: nelite
7: SA—Improve elements of *s*_*t*_ with simulated annealing algorithm. Parameters: niterSANN, stepSANN
8: Elitism—Put *s*_*t*_ in *S*_*t*_,
9: **repeat**
10: Crossover—Randomly pick two solutions in *S*_*t*_. Obtain a recombination of these two solutions.
11: Mutation—Mutate the solution from the above step with a certain mutation probability and intensity. Parameters: mutprob, mutintensity
12: Insert this solution into *S*_*t*_.
13: **until** *S*_*t*_ has *N*_*pop*_ solutions.
14: **until** Convergence: the achievement of the maximum number of iterations or non-improvement for a prescribed number of iterations. Parameters: niterations, miniterbefstop, tolconv **return** Best Solution.

The parameters of the selection algorithm in TrainSel are: “npop” which is the size of the genetic algorithm population, “nelite” which is the number of elite solutions selected in each iteration, “niterations” which is the maximum number of iterations for the genetic algorithm, “miniterbefstop” is the minimum number of iterations of “no change” before the algorithm is deemed converged, “tolconv” which is the tolerance for determining “no change” in the criteria values, “niterSANN” which is the number of iterations for the SA algorithm, “stepSANN” which controls the speed of cooling of the SA algorithm. Each of these parameters comes with default settings, most of which do not need to be changed by the user for small to medium-sized optimization problems. For larger problems increasing “niterations” and “niterbefstop” parameters will usually suffice. We have done some experimentation with the default settings of the remaining parameters (and with relatively large values for “niterations” and “minitbefstop”) algorithm in several problems with different complexities where the true solution was known. The results from these convergence experiments are provided in Supplementary Figure 1. The user can use these figures to guess initial estimates for these two parameters for their problems. After the run of the algorithm, the best way to decide if the algorithm has worked is by checking the flattening of the objective function values during the final iterations.

In most applications of STP, the ordering of selected samples in the TRS will not be important and therefore only one instance of each individual is required for TRS sample; we refer to this case as an unordered set (UOS). In certain cases, the order of the sample will be important but again only one instance of each individual is required, we refer to this case as ordered set (OS). The cases where we allow more than one instance of each individual is referred to as unordered multiset (UOMS) and ordered multiset (OMS). TrainSel allows users to specify which of these types of sets the optimization problem falls into. An application of the use of finding optimal ordered sets is the design of a blocked experiment where we care about the design of the experiment, i.e., the assignment of individuals to different blocks, in addition to selecting which individuals to include in the study.

The search algorithm in TrainSel is not guaranteed to find globally optimal solutions, i.e., the solutions obtained by any run of TrainSel may be sub-optimal, and different solutions can be obtained given different starting conditions and optimization parameters. Another layer of safety can be obtained if the algorithm is started from multiple initial conditions, and the best of all the runs is selected as the final solution.

Numerous other algorithms have been proposed for the optimal subset selection problem, many of them are heuristic exchange type algorithms (Fedorov, 1972; Mitchell, 1974; Nguyen and Miller, 1992; Rincent et al., 2012; Isidro et al., 2015). In exchange type algorithms, new solutions are obtained by adding a sample unit and removing another at a time (some exchange algorithms might allow the exchange of more than one samples at once), these algorithms are greedy and are only proven to find the best subset for a certain type of design criteria.

### 2.3. Design Criteria

Selection of training populations is an optimal experimental design problem, and the work on the optimal experimental designs has a long and rich history (Smith, 1918; Kiefer, 1959; Fisher, 1960; Fedorov, 1972; Atkinson and Donev, 1992; Pukelsheim and Rosenberger, 1993; Fedorov and Hackl, 2012; Silvey, 2013) and it is not a surprise that many different design criteria have been proposed. These criteria can be categorized into three major groups:

- Parametric design criteria which assume that the experimenter has specified a model before the training data is obtained. These criteria depend on a scalar function of the information matrix for the model parameters that give some indication about the sampling variances and covariances of the estimated quantities by the model. The estimated quantity might be some function of the model parameters or predictions from the model for target individuals. There are many designs obtained by optimizing such criteria are referred to as *A*−, *D*−, *E*−, *G*−, etc… optimal designs (Kiefer et al., 1985). Bayesian design criteria use priors on the parameters of the models to evaluate the utility of designs.
- Nonparametric designs include criteria that are based on distance or similarity measures. For example, the maximin-distance design is a space-filling design that chooses a training population such that the minimum distance among the TRS is maximized (Johnson et al., 1990). Another such design is the minimax design (Johnson et al., 1990) where the training population is such that the maximum of the minimum distances from the training population to the rest of the CS or the TS is minimized. Space-filling designs aim to cover the experimental region with as few gaps or holes as possible. Unlike the parametric design criteria, minimax distance presumes no underlying model and, in turn, is suitable for situations where the model is unknown.
- Multiple design. The choice of an appropriate criterion requires knowledge about the model and what is required from the model. Multiple model optimal experimental design and compound optimization criteria try to overcome the choice issue by combining more than one criteria into one via some type of averaging. Alternatively, we can compare different designs using more than one criteria based on the dominance concept and use multi-objective optimization methods to decide on a certain design from out a set of Pareto optimal designs (Markowitz, 1952, 1968; Akdemir and Sánchez, 2016; Akdemir et al., 2019).

TrainSel allows users to use optimization criteria by letting them write their optimization functions and therefore can be used to search designs based on all of the above categories. Given the multitude of design criteria, this flexibility is one key advantage of TrainSel to its alternatives such as STPGA or TSDFGS.

#### 2.3.1. Built in Criterion: CDmin

The STP involves the selection of TS from CS using optimization criteria. TrainSel is supplemented with a predefined design criterion CDmin which is related to the CDmean criteria in Laloë (1993), Laloë and Phocas (2003), Rincent et al. (2012). The main reason for implementing this design criterion as the only built-in design criterion is due to our specific interest in applying TrainSel to the design of single and multi-environmental GP experiments.

The built-in criterion CDmin depends on the linear mixed models. The linear mixed-effects model for a *n*-dimensional response variable *y*, *n* × *p* design matrix of fixed effects, *n* × *q* design matrix of random effects is defined as:

y=Xβ+Zu+ε;

where ε ~ *N*_*n*_(0, *R*) is independent of *u* ~ *N*_*q*_(0;*G*), *β* ∈ *ℛ*^*p*^, *G* is a *q* × *q* covariance matrix and *R* is a *n* × *n* covariance matrix. The assumptions of the linear mixed-effects model imply *E*(*y*|*X*; *Z*) = *X*β**, $y\sim N_{n}\left(X\beta ;ZGZ^{\prime }+R\right)=N_{n}\left(X\beta ;V\right)$ with *V* defined as *V* = *ZGZ*′ + *R*. For this model, the coefficient of determination matrix (Laloë, 1993; Laloë and Phocas, 2003; Rincent et al., 2012) of $\hat{u}$ for predicting *u* is given by

$$\left(GZ^{\prime }PZG\right)⊘G$$

where *P* = *V*^ −1^ − *V*^−1^*X*(*X*′*V*^−1^*X*)^−1^*X*′*V*^−1^ and ⊘ expresses the elementwise division. The minimum of the selected diagonal elements of this matrix is called the CDmin. The minimum of the coefficient of determination takes on values between 0 and 1, and the designs that give higher values for this criterion are preferred to designs with lower values. The CDmin criterion follows the maximin decision rule, maximizing this criterion amounts to maximizing the utility for the worst case scenario, and it is suitable for making risk averse decisions.

Most authors use the mean of the selected diagonal elements of this matrix as the criterion, this is called the CDmean criterion. We have used CDmin instead of CDmean for several reasons. Firstly, the distribution of CD values along the diagonal for a given *G* matrix includes both the training samples and the remaining samples. The CD values that correspond to the training samples, as expected, form a different cluster (high values of CD) than the cluster of CD values corresponding to the samples that are not selected (low values of CD) and therefore we have a bimodal distribution for the CD values. Secondly, if the aim is to improve the generalization performance of the resulting model we prefer to move the lower part of this distribution to the right, i.e., the maximin decision amounts to improving the worst case CD value in this distribution which leads to the CDmin approach. Thirdly, the purpose of this article is not to compare effect of using different selection criteria but to show that TrainSel can be easily adopted to many different selection criteria.

Alternatively, we could approach the bimodality by restricting the mean measure to be calculated only on the set difference of the CS and the TRS or on a predefined TS. It should be trivial to apply any of these modifications with TrainSel. We stress here that the choice among the many different optimization criteria require thorough analysis, but this is beyond the aims of this paper.

We use two parameterizations of the above mixed model: In the first parameterization, we assume that $G=\sigma_{k}^{2}K$ and $R=\sigma_{e}^{2}I$ where $\sigma_{k}^{2}$ and $\sigma_{e}^{2}$ are the variances of the random terms *u* and *e* correspondingly and *K* is a relationship matrix of the same dimension as *G*. In the second parameterization *G* = *K* ⊗ *V*_*k*_ and *R* = *I* ⊗ *V*_*e*_ where *V*_*k*_ and *V*_*e*_ are covariance matrices that relate to the effects in *u* and *e* using Kronecker structured covariances.

The first model is useful for modeling random effects *u* related by a relationship matrix *K*. The STP for this model involves the selection of a predefined size set from the levels of the random term *u* that also correspond to factor levels in the rows (and columns) of *K* for labeling.

The second model is useful for modeling factor levels that correspond to the rows (and columns) of *K* in several related environments. The covariance of these random effects in several environments is given by *V*_*k*_ and similarly, the covariance of the residual effects in these environments is given by *V*_*e*_. In this case, we want to select predefined sizes of sets from the factor levels that correspond to the rows (and columns) of *K* to be labeled in the corresponding environments.

The purpose of the *X* matrix in the mixed models above is to account for fixed effects. If the rows of the *X* matrix corresponding to the conditions in a given environment are heterogeneous, then, in addition to selecting the levels of the random effect in the TRS, we would like to arrange the training sample optimally to the conditions expressed in the rows of *X*. In these cases, we are looking to identify a TRS that is an ordered subset of the CS. If no *X* matrix is specified or if the rows of *X* are homogeneous within environments the order of the assignments will not matter. In this case, STP involves the selection of an unordered sample as TRS.

### 2.4. Datasets and Applications

In this section, we describe the datasets, simulations, and related analysis. We are testing Trainsel with four applications: The first application deals with STP for GP of hybrid performance, the second with a design of multi-environmental GS experiment. The third application deals with STP for an image recognition problem. Our final application on splines regression entails simultaneous selection of design points among a set of candidates and allocation of knots through the range of the explanatory variables.

### 2.5. Application 1: Wheat Data for Hybrid Performance Prediction

This dataset was published in Liu et al. (2016) and was used in a similar context in Guo et al. (2019). The genetic dataset included the marker data (90 k SNP array based on an Illumina Infinium genotyping platform) for 135 elite winter wheat individuals adapted to Central Europe. A total of 1, 604 F1 hybrids were generated in a factorial crossing scheme with 120 inbred individuals serving as female and 15 inbred individuals serving as male parents.

All genomic data for the wheat data for hybrid performance prediction application were obtained from the Dryad Digital Repository (doi: 10.5061/dryad.461nc). All related phenotypic data were obtained from the Digital Repository (doi: 10.5447/IPK/2016/11). Marker information for the hybrids was deduced from the parental individuals.

All individuals were evaluated in up to six environments. The adjusted means over environments for each of the 1, 604 F1 hybrids for 7 traits (gluten content, kernel hardness, protein content, SDS volume, starch content, test weight, 1, 000-kernel weight) were treated as the labels for the traits.

After removing the hybrids that came from parents with partial phenotypic data, we were left with 795 hybrids (full factorial crosses between 15 males and 53 females with complete phenotypic data). We have complete phenotypic data for all of these 795 hybrids in this application. Nevertheless, in practice, the evaluation of each of the hybrids involves making the cross between the corresponding parents and evaluating them in phenotypic trials, which are time-consuming and expensive. It is, therefore, desirable to reduce the costs involved in the generation and phenotypic evaluation by using a subset of all possible hybrids in the experiments and to use the data generated from these experiments for training genomic prediction models to make inferences about the phenotypic performance of untested hybrids.

In this application, we examine STP for hybrid performance prediction, i.e., we would like to select a prespecified size subset (50, 75, 100, 200 hybrids) of all possible 795 hybrids for training and use the phenotypic data from the TRS to predict the performance of the remaining hybrids. The TRSs were determined either by TrainSel using the CDmin criterion or by random sampling (repeated 30 times). The remaining hybrids were used as the TS where the prediction accuracies were evaluated using the correlation or the mean squared error between the predicted genotypic values and the observed phenotypes.

We only used the additive effects when calculating the CDmin criterion values through use of an additive relationship calculated from the marker scores. It is possible to include other effects such as dominance by supplementing the additive effects matrix with a dominance relationship matrix.

### 2.6. Application 2: Wheat Data for Multi-Environmental GS Experiment Design

We have obtained this dataset from https://triticeaetoolbox.org/wheat. The genotypic data included 989 individuals genotyped for 24, 740 markers. All of these individuals had complete phenotypic data on plant height and stripe rust severity from three environmental trials. Using this data we have performed a cross-validation experiment where we explored the potential of STP for the multi-environmental design of GS experiments. We varied the number of overlapping individuals between the environments intending to see the effect on the predictive ability for the untested individuals.

We start each replication of the experiment by randomly selecting 240 individuals as the CS and the remaining individuals as the TS. Given the candidate individuals, we assume would like to construct an experiment in tree environments each of which can accommodate a fixed number of individuals (20, 40, 60, 80). To see how the replication affects the maximum CDmin values we also restrict the total number of individuals in the whole experiment to multiples of 1.2, 1.5, 2, 2.5, 3 of the number of individuals in each environment. Note that, restricting the total number of individuals to a multiple of 1.2 of the number of individuals allowed in each of the environments correspond to almost total replication (we did not use a factor of 1 because this value corresponds to a different type of combinatorial problem), on the other hand, a multiple of 3 corresponds to no replication, the intermediate values allow some amount of replication. We have assumed that the covariance of genotypic values between all trials pairs were 0.7 and we have assumed that the residuals were independent within and between trials. Besides, we have assumed that the heritabilities of both experiments were the same and equal to 0.5. We repeated this experiment 15 times and for each replication, we record the maximum CDmin value obtained and we also check the accuracy of the model in the TSs by calculating the correlation of the trait values in the TS and corresponding predictions from models based on different TRSs.

### 2.7. Application 3: MINST Datasets for Image Recognition

Image classification refers to the task of predicting the kind of objects in images. To train image classification models we need labeled images as training data. In this context, the purpose of STP would be to identify a subset of images to be labeled from out of a larger set of images.

In this application, we used a standard image classification data, the MINST fashion dataset, obtained using the “tf.keras.datasets” module, which consists of 28 × 28 grayscale images of 70, 000 in 10 categories. The original data is split into two parts, the training set has 60, 000 images and the test set has 10, 000 images. In both the training and test datasets, the different classes were equally represented.

We performed the following experiment with this dataset: We started each replication of the experiment by identifying 1, 000 samples at random from the original training set of size 60, 000 as candidates. The number of samples from each class in the CS were arbitrarily set as 500, 450, 400, 350, 300, 250, 200, 150, 100, and 50 to assure an unbalanced CS. We chose a TRS of 100 or 200 samples out of the CS using TrainSel with the maximin distance criterion and using the distances among the 794 image features of samples in the CS. In addition, 100 random samples of sizes 100 and 200 were taken from the same CS as random TRSs. For each TRS, we recorded the entropy for the class distributions in the TRSs, the loss, and the accuracy for the predictions in the TS. We used the same 4-layer convolutional deep neural network prediction model for all the TRSs, these models were trained using the Keras R package (Allaire and Chollet, 2018). This experiment was repeated 50 times.

### 2.8. Application 4: STP for Splines Regression

Spline regression is a commonly used regression technique for modeling nonlinear relationships between a continuous response and continuous explanatory variables. In this technique the ranges of the explanatory variables are divided into bins using points which are called knots and the response is modeled with a piecewise polynomial with a set of extra constraints (continuity, continuity of the first derivative, and continuity of the second derivative) at the knots.

A commonly used form of splines, namely the natural cubic splines, uses cubic segments. The model for a natural cubic spline that relates the response *y* to the input variable *x* can be expressed as

$$y=\beta_{0}+\beta_{1}x+\beta_{2}{\left(x-k_{1}\right)}_{+}+\beta_{3}{\left(x-k_{2}\right)}_{+}+...+\beta_{6}{\left(x-k_{p}\right)}_{+}+\sigma_{\varepsilon }^{2}$$

where

$${\left(x-k\right)}_{+}=\{\begin{array}{l} 0,\text{ if }x<k\\ x-k,\text{ if }x\geq k \end{array}$$

and *k*_1_, *k*_2_, …, *k*_*p*_ are the knot positions that are to be specified as hyper-parameters. Due to this dependence the model matrix for this model will be written as *X*(*k*). The qubic spline is a linear model, therefore, the formula for D-optimality criteria for this model can be expressed as *D*(*k*) = |*X*(*k*)′*X*(*k*)| and its value depends on the choice of the knots. A “good” design maximizes the value of this function, i.e., we need to select the design points and also find the best knots for the selected set of design points.

In this simulation exercise, we show that we can simultaneously pick a TRS of design points out of a set of candidates and set the knot positions using TrainSel, i.e., we want to select a set of *x* values from a set of given candidates and find values of *k*_1_, *k*_2_, …, *k*_*p*_ that maximizes *D*(*k*). Just like in other supervised learning scenarios, we assume we have no access to the values of the response apriori, their values will be observed only in the TRS and these along with the selected optimal knots will be used to fit the cubic spline model. The model will be used in the prediction of the response and the predicted response values in the CS will be compared to the true value of the response (the function value at *x*) by calculating mean squared errors. The results obtained by the optimization approach will be compared to the same size random sample of *x* selected from the CS and with the standard approach that involves placing knots at equally spaced quantiles of the range of the *x* values (Ruppert, 2002) in the CS.

In each replication of the experiment, we started with a 1, 000 candidate *x* values sampled uniformly between 0 and 1. We selected 200 (or 300) *x* values from these candidate values and also determine the placement of 15 knots. Following the benchmark experiments in Ruppert (2002) we generated our response variables from four different functions (namely logit, sine, bump, spahat functions). More details on these functions and the generation of the response values are given in the Supplementary Material. The mean squared error for the predictions from the optimized set with optimized knots and random TRSs with equally spaced quantile knots were compared. This experiment was replicated 30 times.

## 3. Results and Discussion

### 3.1. Application 1: Wheat Data for Hybrid Performance Prediction

**Figure 2:**
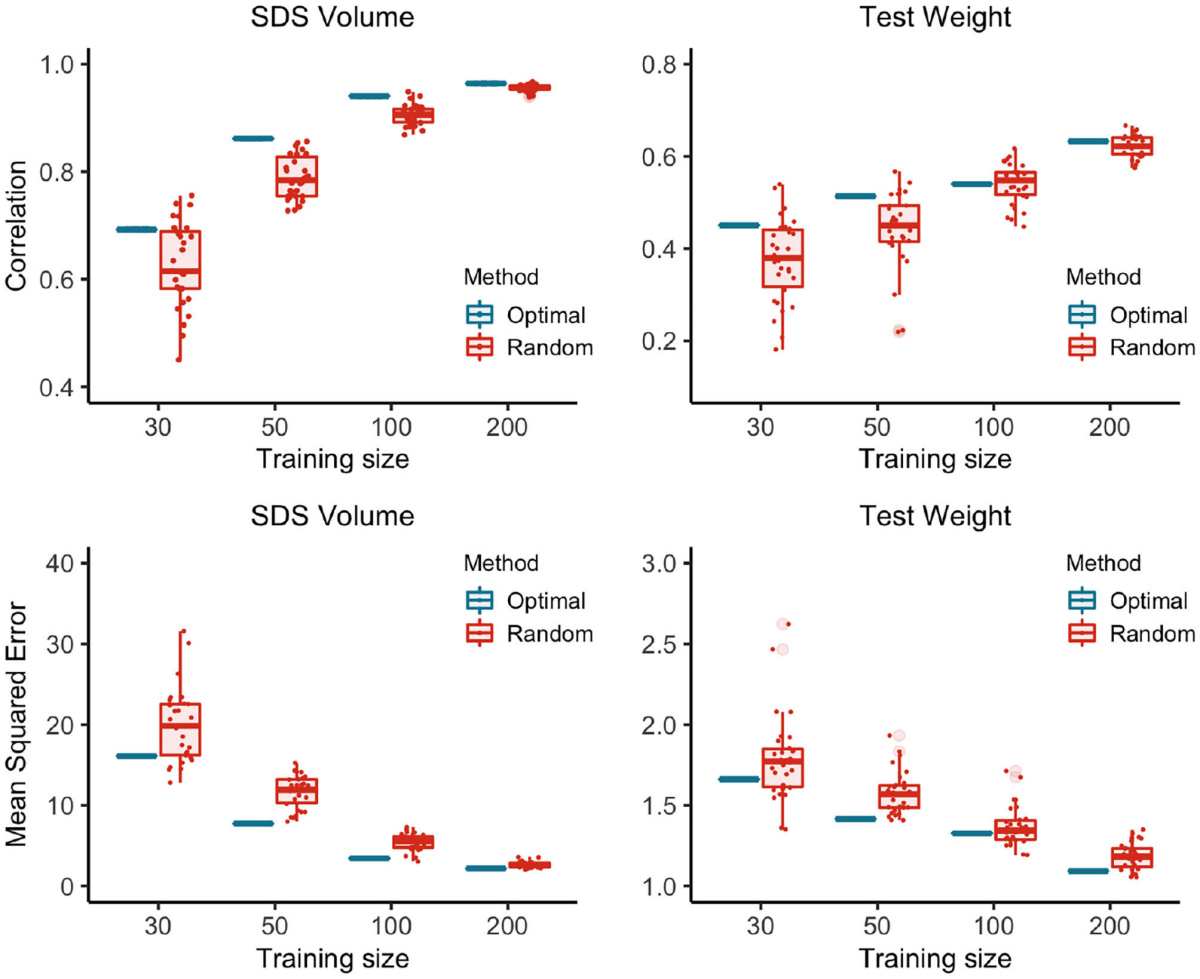
The correlations and the mean squared errors between the predicted and observed trait values of the hybrids in the test data. There is an advantage in using optimized training samples for this dataset. The correlations and mean squared errors between the predicted and observed trait values of the hybrids in the test sets were significantly better for the optimized samples than the correlations and mean squared errors of the predicted and observed for the random samples.

The results of the application on hybrid performance are summarized by the boxplots in Figure 2 for two traits. The results for the remaining five traits were summarized in Supplementary Figure 2. Preliminary analysis with the wheat data indicated that the hybrids selected as training by maximizing the CDmin criterion, provided more accurate prediction models for predicting the remaining hybrids as compared to models based on a random sample of hybrids. The relative efficiency of the optimized samples depended on the number of hybrids selected in the TRS, and also on the trait. Nevertheless, there was a clear optimized trend overall. The relative performance of the optimized TRS to random samples is minimal when the sample size were as low as 50, and it peaked for about sample size of 100, this relative efficiency decreased as the sample size increases. These results indicated that the CDmin criterion was a useful method for selecting wheat hybrids for predictive performance. In our opinion, hybrid prediction problems provide a perfect situation to exploit the STP approaches.

### 3.2. Application 2: Wheat Data for Multi-Environmental GS Experiment Design

When designing a multi-environmental GS experiment, we would like to allocate individuals in environments so that we have a representative sample of individuals in each environment and, at the same time, have genetically similar individuals across environments. Genomic information is not utilized when designing experiments using classical methods such as randomized block design, and therefore, these designs are expected to perform worse than designs that make use of genomic information.

**Figure 3:**
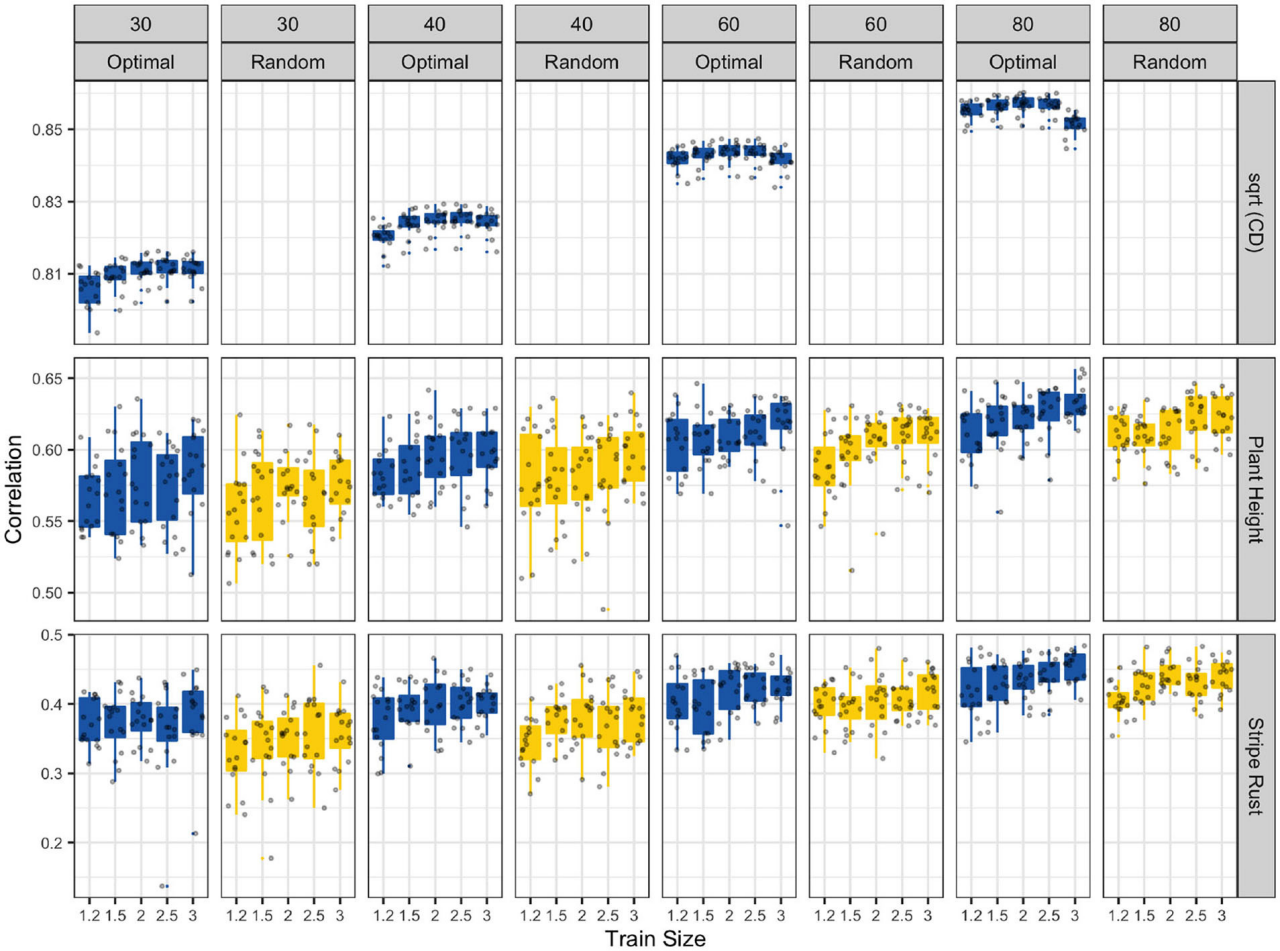
Optimally designed multi-environmental GS experiments can boost prediction accuracies. In the first row, the CDmin values of the optimal samples show that the CDmin values are maximized for the intermediate amount of replication between the experiments. The second and third rows of figure show the attained accuracy for optimal samples and random samples for plant height and stripe rust.

The CDmin values of the optimal samples on the first row of Figure 3 indicate that CDmin values are maximized for intermediate amount of replication between the experiments. Since, the square root of the CD relates directly to the expected accuracy, we can use this information to decide on the size and amount of replication for a multi-environmental GS experiment.

The second and third rows of Figure 3 showed the attained accuracy for optimal samples and random samples for plant height and stripe rust. As we can see the optimal experiments had better accuracy compared to the random experiments at all experiment sizes, levels of replication and for both of the traits. The trends in the observed accuracies for both the random samples and the optimized samples followed the trends observed in the CDmin values in the first row of the Figure 3.

These results demonstrated that optimally designed multi-environmental GS experiments can boost prediction accuracies as compared to randomized block designs. We note here that designing multi-environmental experiments with a large number of candidate individuals can be computationally costly. A useful strategy in these cases involves reducing the size of the candidate set to a manageable size by selecting a optimal subset from the full candidate set using suitable design criterion and using the reduced candidate set in the design of the multi-environmental experiment.

### 3.3. Application 3: MINST Datasets for Image Recognition

**Figure 4:**
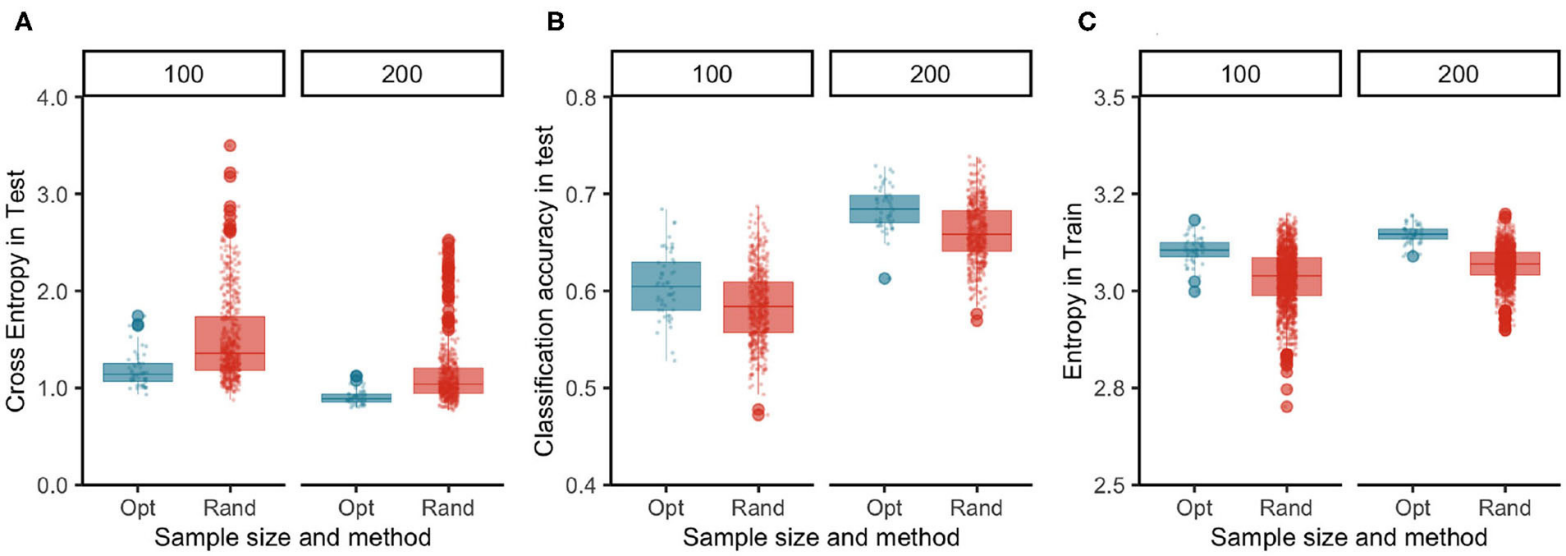
Images selected optimally in the TRS have higher entropy in their label distributions than of the random samples **(C)** and the generalization performance of the model measured by both loss **(A)** and accuracy **(B)** functions in the test dataset indicate that optimally selected samples yield better models than the ones built on random samples.

The results of this experiment are summarized in Figure 4. The TRS identified by TrainSel using the maximin distance criterion had higher entropy in their label distributions on average compared to those of random samples for both TRS sizes (Figure 4). Entropy is a widely used measure for quantifying inhomogeneity, impurity in machine learning applications. The predictions from the models trained on the optimal TRS were on average more accurate and had lower cost as measured by sparse cross-entropy.

Note that, in this application, we have started each replication of the experiment with an unbalanced CS. Entropy is a measure of balance in the label distributions, and entropy of the label distributions in the TRSs selected at random mirrors the unbalance in the CS. In addition, optimally selected samples have higher entropy values meaning that the labels for the samples were more evenly distributed, and this resulted in models with better accuracy, i.e., the percentage of correctly classified examples were higher (Figures 4A–C). In addition, the lower values of the loss function in the test data for optimal samples indicated that the estimates of probabilities used for the classification of observations lead to more confident decisions with more confident class probability estimates.

### 3.4. Application 4: STP for Splines Regression

**Figure 5:**
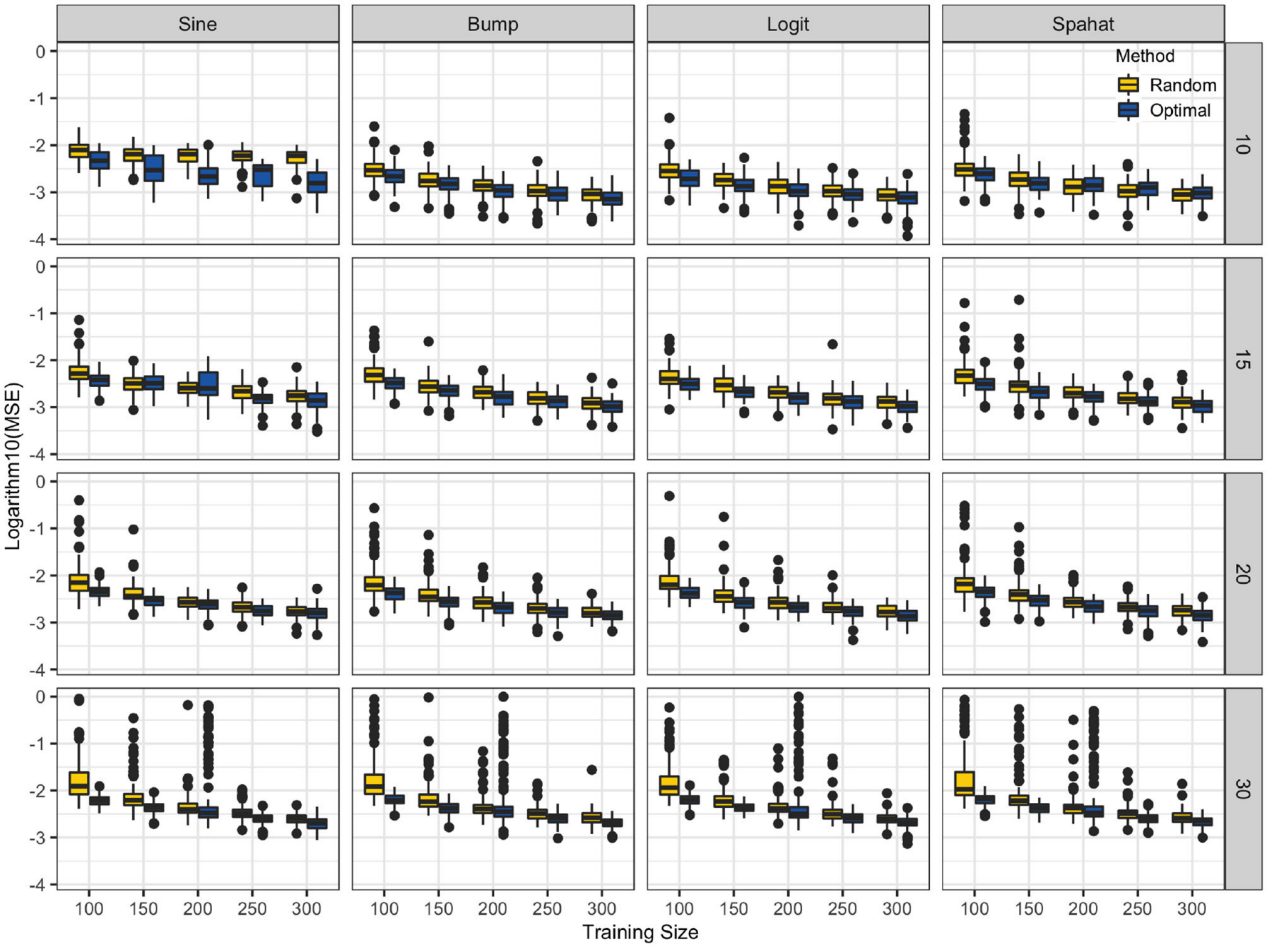
TrainSel spline application. The logarithm of the mean square errors (y-axis) splines models trained on random samples with knots (10, 15, 20, and 30) located at equally spaced sample quantiles. Optimal training size (x-axis) and knots were selected by optimizing the D-optimality criterion for the different number of knots and different sample sizes for a set of functions. At each combination of sample size and the number of knots mean squared errors are lower for the latter approach. Although, in very few cases random sample performed slightly better than the optimized samples, the general trend is in favor of the optimized approach.

The results of the splines experiment are summarized in Figure 5. For all combinations of the number of knots, the number of TRS sizes, the optimally designed experiments where both knot placements and selected samples in the TRS were decided by optimizing the D-optimality criterion have resulted in splines models with lower mean squared error values as compared to the splines models trained on random samples with knots located at equally spaced sample quantiles. This was true for all of the four different response surfaces we have tested.

This example used TrainSel used to optimize a mixed integer optimization problem. Mixed integer programming finds many applications in plant breeding, for instance, it can be used in optimizing sequencing resources (Gonen et al., 2017; Cheng et al., 2020), estimating parental combinations to balance gains and inbreeding (Brisbane and Gibson, 1995; Jannink, 2010; Heslot et al., 2015), or genomic mating (Akdemir and Sánchez, 2016).

## 4. Conclusions

TrainSel provides algorithms for the optimization of mixed-integer problems. It was written with the STP problems in focus. The main use cases are given below:

1. Identifying a TRS from a larger CS for labeling especially when per sample cost of labeling is relatively high.
2. Design of experiments based on any user-defined design criteria or with built-in mixed model-based criteria.
3. Design of single or multi-environmental genomic prediction/selection experiments where the phenotyping is the major constraining factor.
4. TrainSel can also be used in other combinatorial optimization problems. Some examples of such problems include max clique, independent set, vertex cover, knapsack, set covering, set partitioning, feature subset selection (for supervised and unsupervised learning), traveling salesman, job scheduling problems.

The best feature of TrainSel is where we combine training set selection with a particular experimental design, and this option has not been implemented in any other STP software.

Reasons for using this package are as follows:

1. Most of the existing STP or statistical design software (such as TSDFGS, AlgDesign; Wheeler, 2004) will optimize only a few built-in optimization criteria. You can use TrainSel easily with your own design criteria.
2. Existing STP or statistical design software (such as STPGA, TSDFGS, AlgDesign) will optimize a single criterion at a time, but TrainSel offers an additional better possibility, i.e., we can specify multiple objectives that must be optimized simultaneously.
3. TrainSel uses a memetic evolutionary algorithm which in our experiments achieved better convergence than a simple genetic algorithm which was the basis for STPGA and TSDFGS.
4. The ability to handle ordered or unordered samples, with or without replication, along with several numerical variables to optimize user-defined functions makes this package a flexible general optimization tool.

We have illustrated with several applications that the benefits of using TrainSel in STP problems. These applications were mostly related to GP and GS, however, one of the major claims of this article is that the same techniques can be used for any supervised learning problem where labeling samples is the main bottleneck for obtaining the training data. We have exemplified this with two applications, one in image classification and another one related to spline regression.

## 5. Implementation and Usage

TrainSel is implemented in R with most of the code written in Rcpp. Sample usage is illustrated in the Supplementary and also in the help files within the package documentation. The source code and installation details are provided at https://github.com/TheRocinante-lab/TrainSel.

## Data Availability Statement

Publicly available datasets were analyzed in this study. This data can be found at: referenced in the article.

## Author Contributions

DA: conception and design of the work, R and Rcpp programs, drafting the article, and critical revision of the article. JI: drafting the article and critical revision of the article. SR: critical revision of the article. All authors contributed to the article and approved the submitted version.

## Conflict of Interest

The authors declare that the research was conducted in the absence of any commercial or financial relationships that could be construed as a potential conflict of interest. The reviewer RF-N declared a past co-authorship with one of the authors, DA, to the handling editor.
