# Supplementary material for: TrainSel: An R Package for Selection of Training Populations
Source: Front Genet. 2021 May 7;12:655287. doi: 10.3389/fgene.2021.655287 (PMC8138169; doi:10.3389/fgene.2021.655287)
Supplement: Supplementary file 1 [file Data_Sheet_1.PDF]

Supplementary Material

CONVERGENCE EXPERIMENTS

In each experiment, the maximum value for the optimization criteria is equal to the total number of samples selected in the training population and the total candidate size is twice this value. The results of this experiment are displayed in Supplementary Figure S1. For all tested cases optimizing ordered samples is more difficult than optimization over unordered sets. Similarly blocked sampling, i.e., selecting several subsets from several CSs, is more difficult than selecting a single sample from a single set of candidates. In this example, "niterations" parameter was set to 5000 and the "minitbefstop" parameter was set to 200. The rest of the parameters were set to their default values. The results show that STP can be solved to global (or near-global) accuracy within a very reasonable time frame.

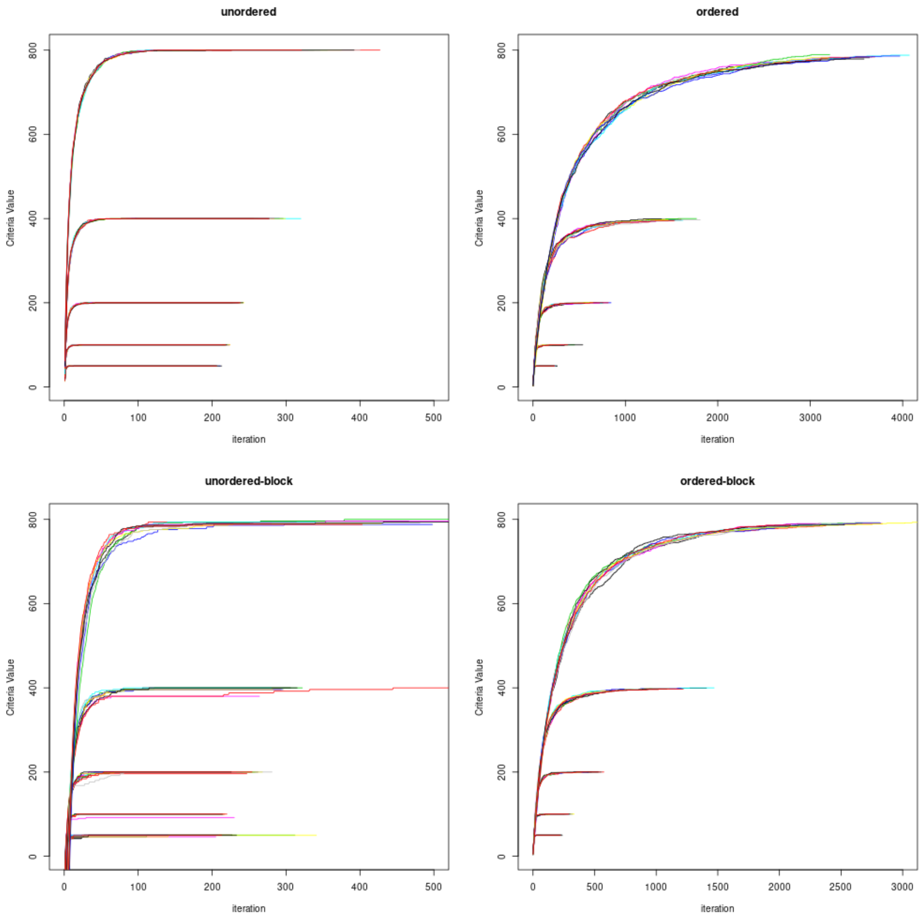

Figure S1. Convergence experiment results. Number of iterations is shown in axis x and the CDmin values on y axis for ordered and unordered experiments with or without blocks.

ILLUSTRATION OF USAGE

In the following, we show how TrainSel can be used for STP for genomic selection.

We first load the library and load the data.

```
library(TrainSel)
#load the data that comes with the package
data(WheatData)
```

The algorithm parameters such as ’npop’, ’niterations’, etc,... can be set using the ’TrainSelControl’ function.

```
#initiate control
control=TrainSelControl()
#change control
control$npop=30 control$nelite=3
```

When using a mixed model based CDmin statistic we need to prepare the data using the ’MakeTrainSelData’ function.

```
#use marker data to prepare data for mixed model based criterion.
TSData<-MakeTrainSelData(M=Wheat.M)
```

We use ’TrainSel’ function to select an unordered sample ("UOS") of size 10 from the first 100 individuals in the dataset (corresponding to the first 100 rows of Wheat.M).

```
out1<-TrainSel(Data=TSData, Candidates=list(1:100), setsizes=c(10),settypes="UOS", control=control)
```

To check for convergence by plotting the objective function values over iterations.

```
plot(out1$maxvec)
```

To use ’TrainSel’ for designing a multi-environmental GS experiment, we first prepare the data by providing the covariance parameters to ’MakeTrainSelData’ function. Here, we assume a covariance of 0.5 between the genetic effects between two environments and assume that environmental variances are equal to 1 in each environment. We assume that the residual variance-covariance matrix is an identity matrix.

```
#use marker data and covariance matrices for environments for ME design
Ve=diag(2);Vk=.5*diag(2)+matrix(.5,nrow=2,ncol=2)
TSData2<-MakeTrainSelData(M=Wheat.M, Ve=Ve, Vk=Vk)
```

We use ’TrainSel’ function to select an unordered sample ("UOS") of size 10 from all the individuals in Wheat.M in the first environment and an unordered multiset ("UOMS") of size 10 from the same individuals.

Note that the 'Candidates' parameter is a list. The first element in this list is '1:nrow(Wheat.M)' and the second element of the list is 'nrow(Wheat.M)+(1:nrow(Wheat.M))'. These refer to the same individuals in different environments.

```
out2<-TrainSel(Data=TSData,Candidates=list(1:nrow(Wheat.M), nrow(Wheat.M)+(1:nrow(Wheat.M))),
setsizes=c(10, 10), settypes=c("UOS", "UOMS"), control=control)
```

HYBRID PREDICTION

The results for the remaining traits for the Wheat data for hybrid performance prediction example.

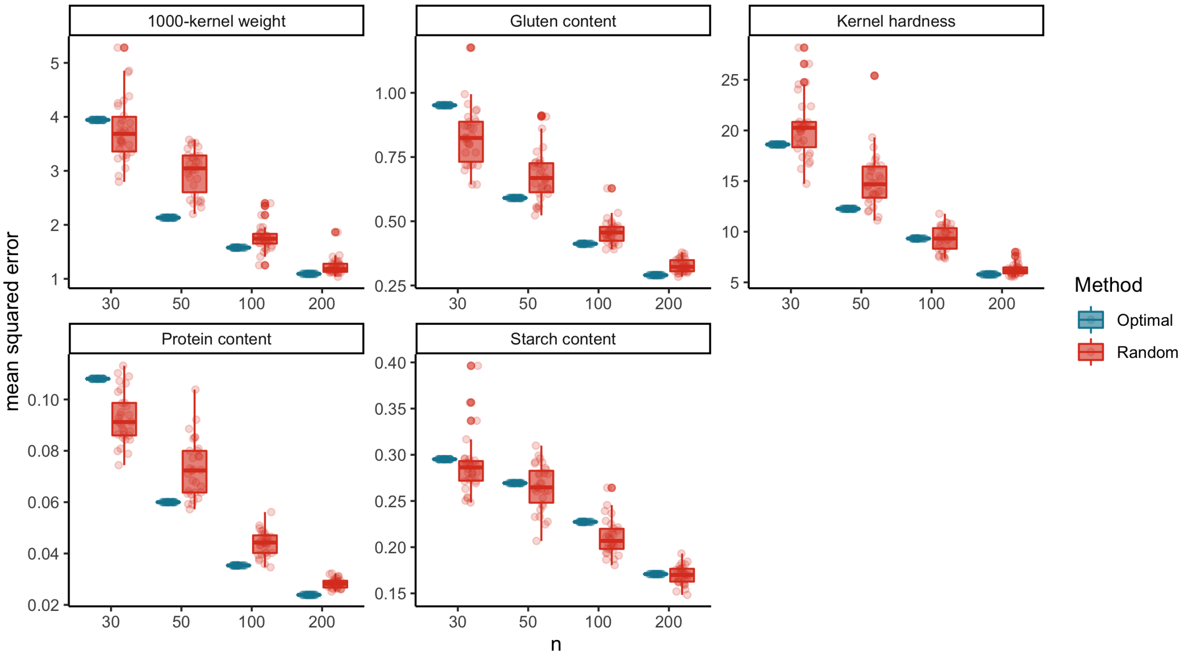

Figure S2. The correlations and the mean squared errors between the predicted and observed trait values of the hybrids in the test data. There is an advantage in using optimized training samples for this dataset.

FUNCTIONS FOR THE SPLINE EXPERIMENT

The response values for the splines application were generated independently according to the following:

- sine function: $y = .5 * sin(2 * pi * (x - .5)) + .5 + e$
- logit function: $y = 1/(1 + exp(-20 * (x - .5))) + e$
- bump function: $y = .4 * ((x - .5) + 2 * exp(-(16 * (x - .5))^2)) + e$
- spahat function: $y = sqrt(x * (1 - x)) * sin((2 * pi * (1 + 2^(- 3/5)))/(x + 2^(- 3/5))) + .5 + e$

where $e \sim N(0, .15^2)$.
